# Towards the prevention of sexually transmitted infections (STIs): Healthcare-seeking behaviour of women with STIs or STI symptoms in sub-Saharan Africa

**DOI:** 10.1136/sextrans-2022-055424

**Published:** 2022-10-06

**Authors:** Abdul-Aziz Seidu, Richard Gyan Aboagye, Joshua Okyere, Collins Adu, Richard Aboagye-Mensah, Bright Opoku Ahinkorah

**Affiliations:** 1 College of Public Health, Medical and Veterinary Sciences, James Cook University, Townsville, Queensland, Australia; 2 Centre for Gender and Advocacy, Takoradi Technical University, Takoradi, Ghana; 3 Department of Family and Community Health, Fred N. Binka School of Public Health, University of Health and Allied Sciences, Hohoe, Ghana; 4 Department of Population and Health, University of Cape Coast, Cape Coast, Ghana; 5 Department of Health Promotion, Education and Disability Studies, Kwame Nkrumah University of Science and Technology, Kumasi, Ghana; 6 Municipal Education Office, Ghana Education Service, Nkawie, Ghana; 7 School of Public Health, Faculty of Health, University of Technology Sydney, Sydney, New South Wales, Australia

**Keywords:** WOMEN, SEXUAL HEALTH, Sexual Behavior, PUBLIC HEALTH

## Abstract

**Objective:**

Sexually transmitted infections (STIs) constitute major public health problems because of their prevalence and contribution to mortality and morbidity worldwide. Healthcare seeking for STIs plays a significant role in the global prevention of STIs. We examined the prevalence and factors associated with healthcare seeking for STIs or STI symptoms among women in sub-Saharan Africa (SSA).

**Methods:**

Data on 38 394 women of reproductive age from the most recent Demographic and Health Surveys of 28 countries in SSA were analysed. Percentages were used to summarise the prevalence of healthcare seeking for STIs or STIs symptoms. The factors associated with healthcare seeking for STIs or STI symptoms were examined using multilevel binary logistic regression analysis. We presented the results using adjusted odds ratios (aORs) with 95% confidence intervals (CIs).

**Results:**

Overall, the proportion of women with STIs or STI symptoms who sought healthcare was 66.1%, with the highest and lowest proportion found in Liberia (85.6%) and Ethiopia (37.9%) respectively. The likelihood of seeking healthcare for STIs or STI symptoms increased with increasing wealth quintile and level of education. Working women, older women, cohabiting women, women with comprehensive HIV/AIDS knowledge, women exposed to mass media, those who had no barrier to healthcare access, and those covered by health insurance had greater odds of seeking treatment for STIs or STI symptoms. On the contrary, the odds of seeking treatment for STIs or STI symptoms was lower among married women and women who lived in rural areas.

**Conclusion:**

The findings of the study call for strengthening of policies, programmes, and interventions geared towards improving thehealthcare-seeking behaviour of women with STIs, taking into consideration the factors identified in this study.

**WHAT IS ALREADY KNOWN ON THIS TOPIC**

- Some women in sub-Saharan Africa with self-reported symptoms of STIs do not seek treatment due to inhibitions and taboos surrounding sexual and reproductive health.

**WHAT THIS STUDY ADDS**

- Overall, 66 out of 100 women who had STIs or showed STI symptoms sought healthcare with the highest and lowest proportions in Liberia (85.6%) and Ethiopia (37.9%).
- The likelihood of seeking treatment for STIs or STI symptoms increased with wealth quintile and level of education.

**HOW THIS STUDY MIGHT AFFECT RESEARCH, PRACTICE OR POLICY**

- The provision of comprehensive sexuality education for younger women (15–19 years) may improve their healthcare-seeking behaviour. Governments in various sub-Saharan African countries would need to create community-based health centres, especially in rural areas where access to a health institution is sometimes difficult.

## Introduction

Sexually transmitted infections (STIs) have been recognised as major public health issues because of their incidence, prevalence, and contribution to mortality and morbidity.^1^ The intricacy of elements involved in modifying sexual behaviour,^2^ the stigma attached to having STIs,^3^ accessing sexual healthcare services,^4^ and moral objections to teaching sex and relationship education in schools^5^ are all issues that obstruct prevention attempts. The efficiency of interventions to lower STI prevalence is unknown, and the cost-effectiveness of STI prevention is a hurdle to political support.^1^ Estimates from the World Health Organization (WHO) indicates that every day, more than 1 million people acquire bacterial STI (gonorrhoea, syphilis, chlamydia and trichomonas) across the globe.^6^

Part of the issue of identifying and controlling STIs is quantifying the proportion of these infections that go undetected and untreated.^7–9^ More than 1 million people worldwide contract STIs every day, making STIs a substantial health burden.^10^ STI prevention and control provide a wide range of benefits and help to achieve the Sustainable Development Goals (SDGs) of providing sexual and reproductive healthcare, eliminating infant mortality, and combatting infectious illness.^8^ In 2016, the WHO published the Global Health Sector Strategy on STIs, with the goal of ending the STI epidemic by 2021, but its achievement remained inconclusive.^11^ Using strong disease surveillance systems, this method creates worldwide targets for tracking progress. The Global Health Sector Strategy on STIs calls for a 90% reduction in gonorrhoea infections, a 90% reduction in syphilis cases, and 50 or fewer occurrences of congenital syphilis per 100 000 live births in 80% of countries by 2030.^11^

STIs place a significant financial strain on the healthcare system. STIs without HIV are consistently among the most common reasons for individuals’ visit to health facilities.^12^ STIs are the main cause of disability-adjusted life years lost for reproductive-age women in low-income and middle-income countries, particularly in sub-Saharan Africa (SSA), behind maternal causes and HIV.^5^ Individuals and communities in poor nations suffer significant productivity losses as a result of STIs.^13^ This has a disproportionate negative impact on women’s health and social well-being by limiting their reproductive potential.^14^

In SSA, there is an unsteady distribution of sexual and reproductive health services.^15^ Women with self-reported symptoms of STIs do not seek treatment due to inhibitions and taboos surrounding sexual and reproductive health.^16^ Report from a study showed that the majority of women with STIs do not seek treatment at health institutions and instead use self-prescribed medications.^16^ Other studies revealed that wealth index, educational status, and working status are associated with increasing healthcare-seeking behaviour for STIs.^4 17 18^

Previous studies conducted in Ethiopia^4 12^ focused on STI-related healthcare-seeking behaviour and associated factors among reproductive age women. However, there has not been any study that has looked at the phenomenon more broadly in SSA. In view of this, the current study examines the predictors of healthcare-seeking behaviour among women with STIs or STI symptoms in SSA. Findings from this study will inform strategies and policies aimed at improving the acceptability and accessibility of STI care services in SSA.

## Methods

### Data source and study design

A cross-sectional analysis was conducted using data from the most recent Demographic and Health Survey (DHS) published from 2010 to 2020 of 28 countries in SSA. Countries were included in the study if their datasets had the variables of interest in this study. In our study, data were extracted from the women’s file (Individual Recode file). The DHS, according to Corsi *et al*,^19^ has been conducted in over 85 low-income and middle-income countries around the world since its inception in 1984. A two-stage cluster sampling technique was used to sample respondents for the survey. The DHS collected data on health indicators such as STIs or STI symptoms from respondents using a standardised questionnaire.^19^ The study included a total of 38 394 women of reproductive age (15–49 years) who reported STIs or STI symptoms and had complete data on the variables of interest in this study. The description of the study sample can be found in table 1. The dataset was obtained freely from https://dhsprogram.com/data/available-datasets.cfm.

**Table 1:** Description of study sample

S/N country	Survey year	Weighted N	Weighted %
1. Burkina Faso	2010	1239	3.2
2. Benin	2018	803	2.1
3. Burundi	2016–17	1221	3.2
4. DR Congo	2013–2014	1836	4.8
5. Congo	2013	1662	4.3
6. Cote d’Ivoire	2011–2012	1417	3.7
7. Cameroon	2018	1715	4.5
8. Ethiopia	2016	264	0.7
9. Gabon	2012	1388	3.6
10. Ghana	2014	1627	4.2
11. Gambia	2019–2020	922	2.4
12. Guinea	2018	1827	4.8
13. Kenya	2014	597	1.6
14. Comoros	2012	209	0.5
15. Liberia	2019–2020	2631	6.9
16. Lesotho	2014	693	1.8
17. Mali	2018	1936	5.0
18. Malawi	2015–2016	2788	7.3
19. Nigeria	2018	3925	10.2
20. Niger	2012	305	0.8
21. Namibia	2013	596	1.5
22. Sierra Leone	2019	2356	6.1
23. Senegal	2010–2011	994	2.6
24.Chad	2014–2015	188	0.5
25. Togo	2013–2014	1062	2.8
26. Uganda	2016	3146	8.2
27. Zambia	2018	488	1.3
28. Zimbabwe	2015	558	1.4
All countries	2010–2020	38 394	100.0

## Variables

### Outcome variable

The study’s outcome variable was healthcare-seeking behaviour for STIs or STI symptoms. With this, the respondents were first asked the question ‘Have you had STI or symptoms of an STI (a bad-smelling, abnormal discharge from the vagina or a genital sore or ulcer) in the 12 months before the survey?’. The response options were ‘yes’ and ‘no’. Those who responded yes were further asked the question ‘When you had the infection, did you seek any kind of advice or treatment?’. The response categories were ‘yes’ and ‘no’. We coded the responses as ‘0=no’ and ‘1=yes’ in the final analysis. This coding was informed by literature that used the DHS dataset.^4 20^

### Explanatory variables

We included a total of 14 explanatory variables, grouped into individual-level and contextual-level variables. The individual-level variables were women’s educational level, current working status, age, marital status, frequency of watching television, frequency of listening to radio, frequency of reading newspaper/magazine, comprehensive HIV/AIDS knowledge, national health insurance coverage, getting medical help for self: getting money needed for treatment, and getting medical help for self: distance to health facility. The contextual-level variables were wealth index, place of residence, and geographical subregions. Previous studies^4 20^ guided the selection of the explanatory variables.

### Statistical analyses

**Figure 1:**
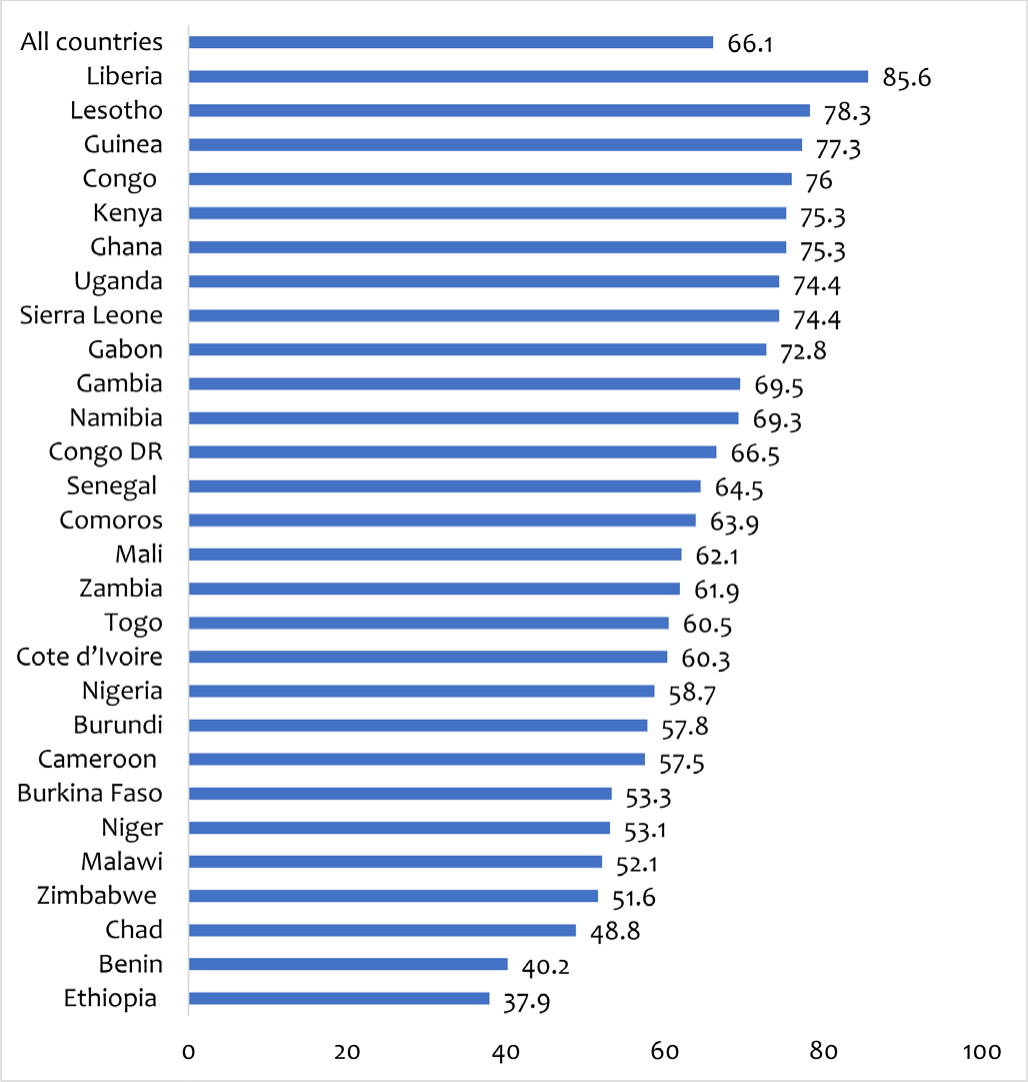
Proportion of women in sub-Saharan Africa who sought for healthcare for STIs or STI symptoms.

We carried out the data analyses using Stata version 16.0. We employed both descriptive and inferential analyses in this study. Descriptively, percentages were used to summarise the proportion of healthcare-seeking behaviour for STIs or STI symptoms (figure 1). We used chi-square test of independence to check for the distribution of healthcare-seeking for STIs or STI symptoms across the explanatory variables (table 2). In the inferential analysis, multilevel binary logistic regression was adopted, relying on four models (Model O–III) to examine the predictors of healthcare-seeking behaviour for STIs or STI symptoms. Model O showed the variance due to the clustering of the primary sample units. Models I and II were fitted to contain the individual and contextual level variables respectively. Model III consisted of all the explanatory variables against the outcome variable. Adjusted odds ratios (aORs) with their respective 95% confidence intervals (CIs) were used to present the results of the regression analysis in a tabular form (table 3). We checked for model fitness and comparison using Akaike’s information criterion (AIC). The model with the smallest AIC value was selected as the best-fitted model for interpretation and discussion. For the regression analysis, statistical significance was set at p<0.05. In all the analyses, we applied the women’s sample weights to obtain unbiased estimates based on the DHS guidelines. To account for a complex sampling structure, the Stata survey command ‘svy’ was employed. The manuscript was written following the Strengthening Reporting of Observational Studies in Epidemiology guidelines.^21^

**Table 2:** Bivariable results of the distribution of healthcare-seeking behaviour for STIs or STI symptoms across the explanatory variables

Variable	Weighted N	Weighted %	Sought advice/treatment	
			Yes (%)	P value
Women’s age (years)				<0.001
15–19	4359	11.4	55.1	
20–24	8500	22.1	66.5	
25–29	8437	22.0	69.3	
30–34	6601	17.2	69.4	
35–39	5185	13.5	67.3	
40–44	3245	8.4	64.4	
45–49	2067	5.4	63.5	
Marital status				<0.001
Never married	7374	19.2	68.7	
Married	20 879	54.4	63.4	
Cohabiting	6473	16.8	71.0	
Widowed	714	1.9	64.1	
Divorced	834	2.2	65.5	
Separated	2120	5.5	69.4	
Educational level				<0.001
No education	10 622	27.7	56.8	
Primary	10 899	28.4	63.3	
Secondary	14 453	37.6	72.5	
Higher	2419	6.3	81.6	
Current working status				<0.001
Not working	12 421	32.4	64.8	
Working	25 973	67.6	66.7	
Frequency of watching television				<0.001
Not at all	18 853	49.1	59.9	
Less than once a week	5988	15.6	68.8	
At least once a week	13 553	35.3	73.5	
Frequency of listening to radio				<0.001
Not at all	14 111	36.8	60.7	
Less than once a week	8999	23.4	68.6	
At least once a week	15 284	39.8	69.6	
Frequency of reading newspaper or magazine				<0.001
Not at all	30 094	78.4	63.6	
Less than once a week	4739	12.3	74.2	
At least once a week	3561	9.3	76.1	
Comprehensive HIV/AIDS knowledge				<0.001
No	22 398	58.3	63.0	
Yes	15 996	41.7	70.4	
Getting medical help for self: getting money needed for treatment				<0.001
Big problem	20 735	54.0	62.7	
Not a big problem	17 659	46.0	70.1	
Getting medical help for self: distance to health facility				<0.001
Big problem	13 972	36.4	61.1	
Not a big problem	24 422	63.6	69.0	
Covered by health insurance				
No	34 981	91.1	64.9	
Yes	3413	8.9	78.7	
Wealth index				<0.001
Poorest	5558	14.5	52.1	
Poorer	6755	17.6	59.2	
Middle	7430	19.3	63.7	
Richer	8798	22.9	70.8	
Richest	9852	25.7	76.3	
Place of residence				<0.001
Urban	18 262	47.6	73.8	
Rural	20 132	52.4	59.1	

*P values are from χ test.

**Table 3:** Fixed and random effect results of factors associated with healthcare-seeking behaviour among women with STIs or STI symptoms

Variable	Model O	Model I aOR (95% CI)	Model II aOR (95% CI)	Model III aOR (95% CI)
Fixed effect model				
Women’s age (years)				
15–19		1.00		1.00
20–24		1.60*** (1.44 to 1.77)		1.57*** (1.42 to 1.74)
25–29		1.95*** (1.75 to 2.17)		1.89*** (1.70 to 2.10)
30–34		2.05*** (1.83 to 2.29)		1.97*** (1.76 to 2.20)
35–39		1.95*** (1.74 to 2.19)		1.85*** (1.65 to 2.08)
40–44		1.83*** (1.61 to 2.08)		1.75*** (1.53 to 1.99)
45–49		1.79*** (1.54 to 2.09)		1.72*** (1.47 to 2.00)
Marital status				
Never married		1.00		1.00
Married		0.85*** (0.78 to 0.93)		0.90* (0.82 to 0.99)
Cohabiting		1.11 (1.00 to 1.23)		1.22*** (1.10 to 1.35)
Widowed		0.89 (0.73 to 1.10)		0.96 (0.78 to 1.18)
Divorced		0.91 (0.74 to 1.11)		0.99 (0.81 to 1.22)
Separated		0.99 (0.85 to 1.15)		1.09 (0.94 to 1.27)
Educational level				
No education		1.00		1.00
Primary		1.27*** (1.18 to 1.37)		1.34*** (1.23 to 1.45)
Secondary		1.66*** (1.52 to 1.81)		1.55*** (1.42 to 1.69)
Higher		2.07*** (1.76 to 2.44)		1.82*** (1.54 to 2.16)
Current working status				
No		1.00		1.00
Yes		1.09** (1.02 to 1.16)		1.10** (1.03 to 1.17)
Frequency of watching television				
Not at all		1.00		1.00
Less than once a week		1.19*** (1.09 to 1.30)		1.02 (0.93 to 1.12)
At least once a week		1.31*** (1.21 to 1.42)		1.04 (0.95 to 1.13)
Frequency of listening to radio				
Not at all		1.00		1.00
Less than once a week		1.16*** (1.08 to 1.26)		1.12** (1.04 to 1.21)
At least once a week		1.13*** (1.06 to 1.22)		1.10* (1.02 to 1.18)
Frequency of reading newspaper or magazine				
Not at all		1.00		1.00
Less than once a week		1.10* (1.00 to 1.22)		1.11* (1.01 to 1.22)
At least once a week		1.10 (0.98 to 1.24)		1.11 (0.99 to 1.25)
Comprehensive HIV/AIDS knowledge				
No		1.00		1.00
Yes		1.27*** (1.19 to 1.35)		1.25*** (1.18 to 1.33)
Getting medical help for self: getting money needed for treatment				
Big problem		1.00		1.00
Not a big problem		1.15*** (1.08 to 1.22)		1.12*** (1.05 to 1.19)
Getting medical help for self: distance to health facility				
Big problem		1.00		1.00
Not a big problem		1.16*** (1.09 to 1.24)		1.07* (1.01 to 1.15)
Covered by health insurance				
No		1.00		1.00
Yes		1.57*** (1.40 to 1.76)		1.59*** (1.43 to 1.78)
Wealth index				
Poorest			1.00	1.00
Poorer			1.30*** (1.19 to 1.42)	1.22*** (1.11 to 1.34)
Middle			1.45*** (1.32 to 1.59)	1.28*** (1.16 to 1.41)
Richer			1.84*** (1.66 to 2.04)	1.54*** (1.38 to 1.72)
Richest			2.27*** (2.03 to 2.55)	1.69*** (1.48 to 1.92)
Place of residence				
Urban			1.00	1.00
Rural			0.71*** (0.65 to 0.77)	0.83*** (0.76 to 0.91)
Geographical subregions				
Southern Africa			1.00	1.00
Central Africa			0.67*** (0.55 to 0.80)	0.76** (0.62 to 0.92)
East Africa			0.65*** (0.55 to 0.78)	0.71*** (0.60 to 0.86)
West Africa			0.69*** (0.58 to 0.82)	0.90 (0.76 to 1.08)
Random effect model				
Primary sampling unit variance (95% CI)	0.17 (0.14 to 0.21)	0.14 (0.11 to 0.18)	0.14 (0.11 to 0.18)	0.13 (0.10 to 0.17)
Intraclass correlation coefficient	0.05	0.04	0.04	0.04
Wald χ^2^	Reference	1189.75***	606.75***	1383.81***
Model fitness				
Log likelihood	−25 251.15	−24 235.80	−24 595.53	−24 052.99
Akaike information criterion	50 506.29	48 525.6	49 211.07	48 175.98
Number	38 394	38 394	38 394	38 394
Number of clusters	1333	1333	1333	1333

*P<0.05, **P<0.01, ***P<0.001; 1.00=reference category.

AOR, adjusted OR.

## Results

### Proportion of women in sub-Saharan Africa who sought for healthcare for STIs or STI symptoms

Figure 1 presents the results on the proportion of women in SSA who sought for healthcare for STIs or STI symptoms. Overall, 66.1% of women in SSA sought healthcare for STIs or STI symptoms. Women in Liberia had the highest proportion of healthcare-seeking for STIs or STI symptoms (85.6%). On the other hand, the lowest proportion was found in Ethiopia (37.9%).

### Bivariable analysis of the distribution of healthcare-seeking behaviour for STIs or STI symptoms across the explanatory variables

In table 2, the results from the distribution of healthcare-seeking behaviour for STIs or STI symptoms across the explanatory variables are presented. The results showed that there was a significant difference in the healthcare-seeking behaviour of women with STIs or STI symptoms across all the variables included in this study. The proportion of seeking advice/treatment was high among women aged 30–34 (69.4%), those cohabiting (71.0%), those with higher education (81.6%), those currently working (66.7%), women who watched television at least once a week (73.5%), those who listened to the radio at least once a week (69.6%), women who read magazines/newspaper at least once a week (76.1%), those with comprehensive HIV/AIDS knowledge (70.4%), women who perceived getting money for treatment as not a big problem (70.1%), those who perceived distance to health facility as not a big problem (69.0%), women who were covered by health insurance (78.7%), those in the richest wealth index (76.3%) and among women living in urban areas (73.8%).

### Fixed and random effect analysis of factors associated with healthcare-seeking behaviour among women with STIs or STI symptoms

#### Fixed effects

The likelihood of seeking treatment for STIs or STI symptoms increased with increasing wealth quintile and level of education. Working women, older women, cohabiting women, women with comprehensive HIV/AIDS knowledge, women exposed to media, those who had no barrier to healthcare access and those covered by health insurance had higher odds of seeking health advice/treatment for STIs or STI symptoms compared to women who are currently not working, women aged 15-19, never married women, women without comprehensive HIV/AIDs knowledge, those with barriers to healthcare, and those not covered by health insurance, respectively. On the contrary, the odds of seeking health advice/treatment for STIs or STI symptoms was lower among married women and women who lived in rural areas compared to never married and women in urban areas (table 3).

#### Random effect

Our results show that model III was the model of best fit for showing the factors that predict the healthcare-seeking behaviour of women with STI. The intraclass correlation coefficient (ICC) result (ICC=0.04) indicates that 4% of the variations observed in this study were explained by model III. The proportion of variance in the null model was 0.05, then decreased to 0.04 in model I while remaining at 0.04 in models II and III (table 3).

## Discussion

This study sought to examine the prevalence and predictors of healthcare-seeking behaviour among women with STIs or STI symptoms in SSA. The study revealed that 66.1% of women with STIs or STI symptoms sought for healthcare. Our findings are consistent with related studies conducted in Ethiopia^22^ and Ghana^23^ that showed that more than half of women with STI had sought health advice or treatment. Nevertheless, this proportion varied at the intercountry-level comparison. Liberia reported the highest proportion of healthcare-seeking behaviour, whereas Ethiopia reported the lowest proportion.

Women’s age emerged as a significant factor that predicted the likelihood of seeking health advice/treatment among women with STIs or STI symptoms. Compared with adolescent girls (15–19 years), women aged 30–34 had greater odds of healthcare-seeking behaviour. This finding aligns with those of Sawyerr,^23^ who found that in Ghana, adolescent girls with STIs had lower odds of seeking health advice and treatment. Similar results from Nigeria support our finding that, compared with women younger than age 19, women of older age (20 years and above) had significantly higher odds of seeking health advice/treatment.^17^ Often, women younger than age 19 tend to lack knowledge and awareness about health services as compared with women of older age (20 years and above).^23^ Hence, this could possibly be the reason for our findings. Also, adolescent girls in most sub-Saharan African context are not expected to engage in sexual relationships. Consequently, there is shame and lack of confidence in attempting to seek healthcare for STIs.^23^ Also, adolescent girls are often stigmatised by the larger community and healthcare professionals,^24^ thereby reducing their tendency to seek healthcare for STIs.

Also, our study found that the odds of healthcare seeking for STIs or STI symptoms increases with increasing level of education. Consistent with a preponderance of evidence from Ghana,^23^ Mozambique^25^ and Kenya,^26^ this study found that higher education yielded the highest odds of seeking health advice/treatment. A plausible explanation for this finding could be that formal education provides an avenue for developing and increasing women with STIs knowledge about the disease and the need to seek treatment. Hence, women with higher education are more likely to be exposed to information about the relevance of good healthcare-seeking behaviour and the potential risks associated with refusing or delaying healthcare seeking.

Our study revealed that women with comprehensive HIV/AIDS knowledge are more likely to seek treatment than women without comprehensive HIV/AIDS knowledge. This may be explained from the perspective that women who lack comprehensive HIV/AIDS knowledge are easily swayed to interpreting the disease as a working of witches or spiritual entities.^26^ Such misperceptions and misinterpretations largely caused by a lack of comprehensive HIV/AIDS knowledge could significantly limit women with STIs healthcare-seeking behaviour. Moreover, it is possible that women with comprehensive HIV/AIDS knowledge have greater healthcare knowledge, which translates to a situation whereby they are more likely to seek healthcare for STI symptoms.

Analogous to previous studies, our study found accessibility to be a strong predictor of healthcare-seeking behaviour among women with STIs or STI symptoms. The probabilities of seeking treatment were linked to accessibility in terms of getting the money needed for treatment and distance to health services. Women who said they could receive the money they needed for treatment and that getting to the health facility was not a problem had a higher chance of getting help. This corroborates previous studies from Uganda^27^ and India^28^ that found poor healthcare-seeking behaviour among women with STIs who considered the distance to health facilities to be problematic. This finding epitomises the extent to which community poverty and deprivation affect the healthcare-seeking behaviours of women with STI. The assumption here is that farther distance to health facilities presents an additional cost to women, thereby becoming a disincentive for women with STI to seek treatment. It is therefore not surprising that our findings showed a significant association between health insurance coverage and the likelihood of seeking treatment. Hitherto, getting money needed for treatment was a challenge and impeded many women’s healthcare-seeking behaviour; however, health insurance aids in eliminating or limiting out-of-pocket payments, hence encouraging women with STIs to seek treatment. This supports the findings of a comparable Ghanaian study,^23^ which found that health insurance coverage was substantially correlated with health-seeking behaviour.

Wealth index strongly influenced the healthcare-seeking behaviour of women with STIs or STI symptoms, according to our findings. Women in the middle, richer and richest wealth indexes had considerably higher likelihood of getting therapy than those in the poorest wealth index. This is in line with Sawyerr’s findings,^23^ who found that women in the wealthiest wealth index are more likely to seek medical treatment. Wealth offers women with STI the opportunity to afford healthcare seeking and treatment. Wealth empowers women and provides them with an impetus to make healthcare decisions which is critical to fostering good healthcare-seeking behaviours.^29^ The study also found that the likelihood of seeking treatment was lower among rural-dwelling women as compared with their counterparts in the urban areas. Similar findings have been reported in Kenya^26^ and Ghana.^23^ Women in rural areas often lack access to health facilities and health messages and information, hence making them less likely to seek treatment. The situation is further compounded by the level of poverty in rural areas.^26^

At the regional level, our result found that compared with women in Southern Africa, those in Central and Eastern Africa had significantly lower odds of seeking healthcare advice and treatment for STIs or STI symptoms. The reason for this finding is uncertain. However, both Central and Eastern Africa are hotspots for political and civil unrest as well as terrorism.^30^ Such unrest results in disruption in the health system and healthcare-seeking behaviour of women. Another possible explanation may be due to the level of STI risk perception such as HIV and AIDS and stigma that varies across these regions.^31^ However, our study variables did not include perceived risk and stigma. As such, we are unable to categorically state that these factors account for the regional-level variations in STI healthcare-seeking behaviour.

### Policy implications

Our findings underline the importance of implementing and strengthening existing programmes and treatments that target low-income women, young women (15–19 years) and those without health insurance. The provision of comprehensive sexuality education for younger women (15–19 years) may improve their healthcare-seeking behaviour. Governments in various countries in SSA would need to create community-based health centres, especially in rural areas where access to a health institution is sometimes difficult.

### Strength and limitations

The study’s key strength is the use of a nationally representative dataset with a large sample size. The DHS survey followed best standards in terms of technique, resulting in a high response rate when combined with the employment of professional and well-trained data collectors. Regarding the limitations, the factors associated with healthcare seeking was not disaggregated by individual countries. Therefore, we are unable to show whether the associated factors of STI healthcare-seeking behaviour varied between the countries. Also, the variables used to create STI were self-reported. Therefore, there is likely to be recall and social desirability bias, which may affect the validity of the study findings. Given that the definition of STI was based on a very limited list of STI symptoms in the DHS, there is a possibility of underestimation of the phenomenon among women. Also, we could not determine that healthcare-seeking behaviour for ulcers or sores and discharges in the study. Additionally, we can draw associations but not causal interpretation from the findings.

## Conclusion

Educational level, current work status, marital status, age, frequency of listening to the radio and reading magazines/newspapers, comprehensive HIV/AIDS knowledge,barriers to healthcare (distance and money needed for treatment), health insurance coverage, wealth index, place of residence, and geographical subregions are all factors that influence the healthcare-seeking behaviour of women with STIs or STI symptoms. As a result, it is vital that policies and, programmes aimed at improving the health-seeking behaviour of women with STIs or STI symptoms take these factors into account when planning and implementing them.

## Data Availability

Data are available in a public, open access repository. Data are available upon reasonable request. The datasets used and/or analysed during the current study are available for download https://dhsprogram.com/data/available-datasets.cfm.
